# The Context of COVID-19 at 18 Months in Relation to Depression, Anxiety, Insomnia: The Emerging Role of Post COVID-19 Symptoms

**DOI:** 10.32872/cpe.13243

**Published:** 2025-08-29

**Authors:** Karin C. Brocki, Monica Buhrman, Farzaneh Badinlou, Lance M. McCracken

**Affiliations:** 1 Department of Psychology, Uppsala University, Uppsala, Sweden; 2 Department of Clinical Neuroscience, Karolinska Institutet, Stockholm, Sweden; Friedrich-Alexander-Universität Erlangen-Nürnberg, Erlangen, Germany

**Keywords:** COVID-19, pandemic, mental health, depression, anxiety, insomnia, Post COVID-19 Condition

## Abstract

**Background:**

The COVID-19 pandemic naturally raised concerns about mental health and wellbeing around the world. As time passed, persisting physical and mental symptoms of post COVID-19, referred to as Post COVID Condition (PCC), have become an increasing concern. The aim of this study was to investigate the stability of symptoms of mental ill health in Sweden in the late phase of the pandemic and the prevalence of persistent symptoms post COVID-19 and interrelations between them.

**Method:**

We measured depression, anxiety, and insomnia, through a one-time online survey in Sweden (*n* = 1,482, mean age 47.6 years; 89.5% women) and used correlation and regression analysis to study potential predictors and their interrelations with PCC symptoms.

**Results:**

Compared to our previous study during the pandemic (May – June 2020), a marginal decrease was found for depression (27% versus 30%), a larger decrease for anxiety (16% vs 24%), and an increase for insomnia (45% vs 38%). Persistent symptoms were frequently reported, with 84.5% reporting at least one symptom, and 49.7% attributing one or more of these to COVID-19 infection. A history of poor mental health and COVID-19 related worry appeared as the strongest risk factors for mental ill health. Persistent symptoms also predicted these outcomes.

**Conclusions:**

Based on comparison with pre-pandemic rates, it appears that the pandemic continued to exert a negative impact on mental health in Sweden. Persistent symptoms, associated with COVID-19 exposure, appear common and may represent a vulnerability factor for mental ill health, along with other factors, including history of a mental ill health and specific pandemic worries.

## Highlights

- Mental health problems persist 18 months post-pandemic.
- High rates of depression, anxiety, and insomnia are documented.
- Symptoms of Post COVID-19 Condition appear highly prevalent, notably fatigue.
- Risk factors for persistent symptoms include health history and pandemic-related worries.

## Background

During the COVID-19 pandemic mental health and wellbeing was a concern, especially for the most vulnerable (World Health Organization, 2020; Yu et al., 2021). Global mental health was rapidly studied during the early pandemic, documenting considerable suffering (Mukherjee et al., 2021). Years following the pandemic the question becomes what does the picture look over time? Did mental health and wellbeing worsen, or did people demonstrate resilience and adjust? Tentative evidence suggests that some recovery took place (Manchia et al., 2022; Yarrington et al., 2021). On the other hand, there is also evidence of persistent impacts 6 and 12 months later in COVID-19 survivors (Mazza et al., 2022; Taquet et al., 2021) suggesting long lasting effects. Thus, more careful study is needed to determine what has happened and what to do about it (Gruber et al., 2021; O’Connor et al., 2021; Tegnell, 2021).

During May and June 2020, we conducted a survey in Sweden with the purpose to assess mental health and wellbeing and factors related to these (McCracken et al., 2020). Based on responses from 1,212 adults we found high rates of depression, anxiety, and insomnia, at 30%, 24%, and 38%, respectively. We further found that these outcomes were associated with poor self-rated overall health, a history of mental health problems, presence of COVID-19 symptoms, and specific worries around health and finances (see also Rondung et al., 2021). It is not known whether circumstances improved or declined for mental health, in Sweden or globally, during the years that followed.

An unexpected result from the pandemic is a high rate of Post COVID-19 Condition or PCC (Fernández-de-las-Peñas et al., 2021; Nalbandian et al., 2021). This includes persisting symptoms potentially involving multiple organ systems, including fatigue, breathing problems, cognitive disturbance, problems with the gastrointestinal tract, joints, skin, problems with mood and sleep, and other problems, that follow infection by the coronavirus (Carfì et al., 2020; Hayes et al., 2021; SBU, 2020). The prognosis of persisting symptoms remains unclear, and it appears that additional research and new models of care are needed (Nalbandian et al., 2021). It is unknown how these symptoms correlate with, or perhaps contribute to, mental health outcomes.

The purpose of this study was to repeat our study conducted in Sweden during June 2020 and reassess the state of mental health and wellbeing 18 months following the start of the pandemic using an independent population sample. The outcomes were levels of depression, anxiety, and insomnia as they are sensitive to stressful events and serve as important predictors of both emotional and physical health. The second purpose was to examine the prevalence of PCC (i.e., symptoms lasting for at least six weeks following infection (e.g., physical, and mental dysfunctions and cognitive impairments – see method section for full description) and their relations with mental health outcomes. Additional questions addressed included the identification of risk factors previously associated with health outcomes, such as a previous history of mental health problems and COVID-19 related worry.

## Method

Data collection for this one-time cross-sectional survey in Sweden occurred from June to August 2021. Participants were recruited online via notices on social media and Uppsala University hospital and Uppsala university homepages. It was only required that participants were adults, living in Sweden, and able to respond to survey material online in Swedish. Study data were collected and managed using Research Electronic Data Capture (REDCap), a widely used electronic survey tool hosted locally at Uppsala University (Harris et al., 2009, 2019). Recruitment of participants continued until the rate of recruitment reduced and before the recruitment interval became extended beyond two months.

### Participants

Initially, 1,657 people provided their consent and entered the survey. In general, there was an increase in missing data with each subsequent measure in the survey, due to dropout, as opposed to skipped or missed items. There was a 10.6% non-completion rate of the three mental health outcome measures and the sample size retained was *n* = 1,482. However, sample size varied slightly from analysis to analysis due to small amounts of missing data on other individual variables.

Participant characteristics are included in Table 1. Notably, the percentage of women who participated was 89.5%. Mean age of the total sample was 47.6 years, *SD* = 11.7, and range was 18 to 81. The participants were well educated with 61.1% having completed university or post graduate education. The vast majority of participants were from Sweden or another Scandinavian country, 90.1%. Most were married or in a relationship, 75.0%, or single, 18.3%, and a little less than half had young children, 45.9%. Most participants were working either full or part time, 80.3%, and the next largest category was retired, 6.8%. A substantial proportion reported a history of mental health difficulties, 43.2%. Fewer, 27.9% of the total sample, were currently experiencing these, and 23.9% reported they had a formal diagnosis.

### Measures

Demographic variables included those detailed in Table 1. To further clarify, participants were also asked whether they (a) lived in suburbs, city, or countryside; (b) had children living at home; and (c) the number of people living in their home in total; whether (d) they had received a formal diagnosis of a mental health condition; (e) were experiencing one of these currently; or (f) were suffering with any of the identified conditions placing people at risk of a poor outcome from COVID-19, including age over 70, hypertension, angina, stroke, heart disease, diabetes, cancer, smoking, respiratory disease, and immune suppressant. Specific worries about COVID-19 were assessed with items used in our previous study (McCracken et al., 2020). Here participants rated their worries about their own health, others’ health, their personal finance, world economy, and the future, on a 5-point scale from 1 (Not at all worried) to 5 (Extremely worried). The respondents’ answers to the five worry items were summed up to calculate a total worry score (α = .77).

#### Persistent Symptoms

Persistent symptoms were defined as symptoms lasting for at least six weeks in one or more of 25 different domains. These were based on the PCC report from the Swedish Agency for Health Technology Assessment and Assessment of Social Services (SBU, 2020) based on available literature at the time and reported on 21 December 2020. The following symptoms were included: fatigue, sleeping problems, problems with attention, joint pain, memory difficulties, depression, headache, impaired daily functioning, anxiety, shortness of breath, pins and needles, gut problems, heart palpitations, changes in smell, changes in taste, decreased lung function, chest pain/pressure, cough, nausea, skin changes, appetite loss, sore throat, weight loss, fever, and reduced quality of life. Participants were presented with the following: “Do you have one or more of the following long-term symptoms? With long-term symptoms we mean symptoms that have persisted for at least six weeks”. If the participant answered “Yes” to any of the symptom questions, they were further asked whether they attributed symptoms they experienced to a previous COVID-19 infection.

#### Standardized Measures

Three well-established, widely used, and properly validated measures were utilized to assess depression, anxiety, and insomnia (see previous report for full descriptions; McCracken et al., 2020, 2021). These measures included the Patient Health Questionnaire (PHQ-9; Kroenke & Spitzer, 2002) for depression, the Generalized Anxiety Disorder scale (GAD-7; Kroenke et al., 2007) for anxiety, and the Insomnia Severity Index (ISI; Bastien et al., 2001) for insomnia.

The PHQ-9 scores range from 0 to 21, with recommended cutoff points for depression severity set at 5 (mild), 10 (moderate), 15 (moderately severe), and 20 (severe) (Kroenke & Spitzer, 2002). In addition to the nine items on the PHQ-9, an additional global rating item assesses functional impairment. The GAD-7 scores also range from 0 to 21, with a cutoff score of 10 identified as optimal for sensitivity and specificity (Kroenke et al., 2007). The ISI total score ranges from 0 to 28 and is categorized as follows: absence of insomnia (0-7), sub-threshold insomnia (8-14), moderate insomnia (15-21), and severe insomnia (Morin et al., 2011).

### Statistical Analysis

Data were analyzed using IBM SPSS version 26.0. First, sample characteristics including rates of infection, vaccination, other health-related descriptors, and rates of cases meeting clinical cutoffs for depression, anxiety, insomnia, and persistent physical symptoms were analyzed. Next Pearson correlation coefficients were calculated to identify background or health status factors significantly associated with depression, anxiety, and insomnia scores. Categorical background variables, such as work and relationship status, were dichotomized as labelled, for example, into “out of work” versus not, and “in a relationship” or not. A second set of correlation analyses included depression, anxiety, and insomnia with each of the individual persistent physical symptom reports and the summary of the total number reported, excluding sleep, depression, anxiety, quality of life, and daily functioning, to avoid inflating the correlations. Additional correlation analyses examined relations between participant attributions of symptoms to a COVID-19 infection, this time analyzing the full symptom set again examining whether the symptom was regarded as a direct result of infection with the COVID-19 virus or not. Finally, hierarchical multiple regression analyses were conducted, with depression, anxiety, and insomnia scores as the criterion variables. In these analyses, age, being in a relationship, being out of work, and having above average financial status were included as background variables in the first block of predictors, based on the correlation analyses. The second block of predictors included relevant health status variables, including self-rated physical health, reported mental health history, and a summary score of risk factors for poor COVID-19 outcome. The third block included reported infection with COVID-19. The fourth block included the summary score for COVID-19-related worry. And finally, a selected set of persistent physical symptom reports plus the summary total of symptoms reported was included in the final block of predictors. The included individual symptoms were selected based on having achieved a medium sized correlation or larger with either depression, anxiety, or insomnia in the correlation analyses.

## Results

### Descriptive Statistics

Table 1 includes descriptive statistics on COVID-19 infection and vaccination related data.

**Table 1:** Sample Characteristics (N = 1,657) and Mental Health Results

Variable	*n*	%
Sample Characteristics		
Gender		
Female	1483	89.5
Male	154	9.4
Non-binary	6	0.4
Education		
Pre-secondary	39	2.3
Secondary	326	19.8
University	1206	73.5
Post graduate	71	4.3
Country of Birth		
Sweden	1430	87.1
Other Scandinavian country	49	3.0
Other European country	121	7.4
Other	41	2.6
Domestic Status		
Married	742	45.2
In a relationship	411	25.0
Single	301	18.3
Divorced/separated	99	6.0
Living apart	79	4.8
Widowed	10	0.6
Work Status		
Working full time	1072	65.3
Working part time	247	15.0
Retired	111	6.8
Student	77	4.7
Sick leave	69	4.2
Unemployed	31	1.9
Parental leave	29	1.8
Unpaid work	6	0.4
Self-Rated Economic Status		
Average	762	46.4
Above average	584	35.6
Below average	185	11.3
Much below average	58	3.5
Much above average	53	3.2
Self-Rated Health Status		
Good	595	37.0
Average	568	35.3
Very good	239	14.9
Poor	177	11.0
Very poor	30	1.9
History of a Mental Health Condition		
No	927	56.8
Yes	705	43.2
COVID-19 Vaccine		
Two dose	862	53.6
One doses	407	25.3
Three doses	339	21.1
Infected with COVID-19		
No	784	48.8
Yes, diagnosed	580	36.1
Yes, unconfirmed	242	15.1
Physical Risk Factors^a^		
None	1065	66.5
One	380	23.7
Two	118	7.4
Three or more	39	2.4
Mental Health Outcome Results		
Depression (PHQ-9; *M* = 6.8, *SD* = 5.9)		
Minimal (Range 0 – 4)	630	44.7
Mild (Range 5 – 9)	393	27.9
Moderate (Range 10 – 14)	221	15.6
Moderately severe (Range 15 – 19)	107	7.6
Severe (Range 20 – 27)	59	4.2
Anxiety (GAD-7; *M* = 4.9, *SD* = 4.8)		
None (Range 0 – 4)	810	57.0
Mild (Range 5 – 9)	380	26.8
Moderate (Range 10 – 17)	190	13.4
Severe (Range 15 – 21)	30	2.8
Insomnia (ISI; *M* = 9.4, *SD* = 6.5)		
None (Range 0 – 7)	629	44.5
Sub-threshold (Range 8 – 14)	474	33.6
Moderate (Range 15 – 21)	235	16.6
Severe (Range 22 – 28)	75	5.3

^a^Sum of risk factors: age over 70, hypertension, angina, stroke, heart disease, diabetes, cancer, smoking, respiratory disease, and immune suppressant.

### Mental Health Results

Table 1 includes summary results from the measures of depression, anxiety, and insomnia. Mean values fall in the mild or subthreshold range, and most people fall below the clinical cutoff in each case. On the other hand, 27.4%, 16.2%, and 45.0%, met criteria for clinically significant depression, anxiety, and insomnia, respectively, based on cutoff scores > 10. Further, 13.6% reported some frequency of thoughts of being better off dead or self-harm (PHQ-9 item 9), and 67.3% reported that depression symptoms were associated with some level of difficulty in work, home, or social activities (PHQ-9 item 10). For potential comorbid presentations of these three conditions, 50.6% met criteria for at least one, 25.6% for at least two, and 12.7% for all three. Naturally these measures are intercorrelated, with depression and anxiety scores correlated at *r* = .82, depression and insomnia at *r* = .67, and anxiety and insomnia at *r* = .65.

Data on worries about own health, health of friends or family, own finances, national or international finances, or the future, was provided on a scale from 1 to 5, from not at all worried to extremely worried. The highest area of worry was around health of friends or family, *M* = 2.85, *SD* = 1.25, followed by national or international economy, *M* = 2.68, *SD* = 1.19, and the future, *M* = 2.68, *SD* = 1.30. Worry about own health, *M* = 2.43, *SD* = 1.91, and own finances, *M* = 1.99, *SD* = 1.23, were lowest ranked.

### PCC Results

Overall, 84.5% of survey participants reported at least one persisting COVID-19 symptom from the set of 25 and the mean number of symptoms reported was *M* = 6.5, *SD* = 5.3. At least one symptom was reported by 78.0% of those who reported they were never infected with COVID-19 and 90.5% of those who reported that they had been. These percentages, although both high, were significantly different, χ^2^(1, *N* = 1,552) = 46.3, *p* < .001. With the removal of the symptom items related to sleep, depression, anxiety, quality of life, and daily functioning, high percentages of participants reported at least one of the remaining 20 symptoms, including 73.1% of those who reported never having COVID-19 and 88.2% of those who reported having it, χ^2^(1, *N* = 1,552) = 57.0, *p* < .001.

**Figure 1:**
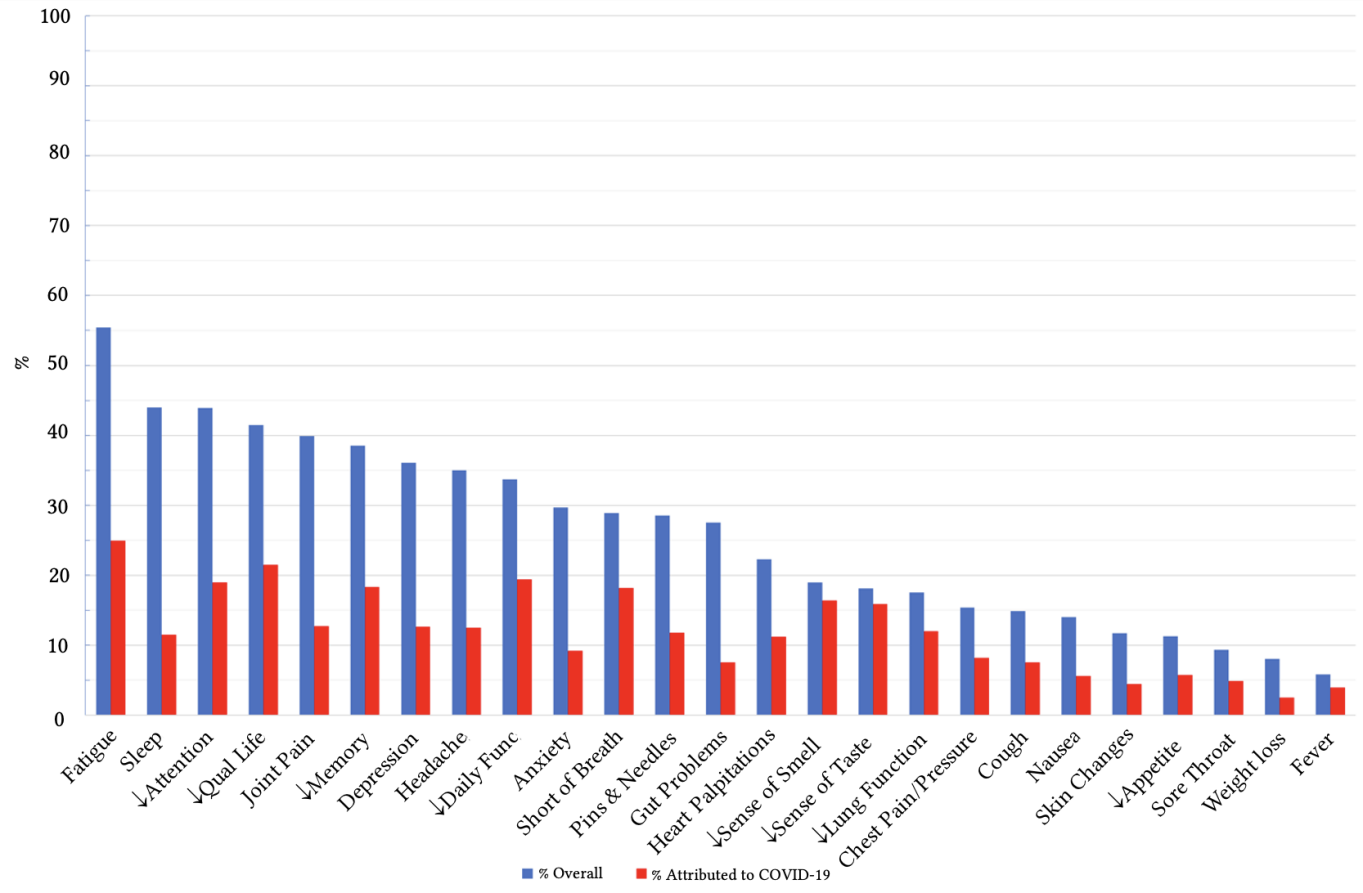
Reported Rates (%) of 25 Symptoms Potentially Linked to COVID-19 *Note.* The downward arrow symbol (↓) indicates a reduction in that domain (e.g., ↓Attention = reduced attention, ↓Daily Func = reduced daily functioning, ↓Appetite = reduced appetite, ↓Qual Life = reduced quality of life).

Figure 1 includes the percentages for persons reporting each of the 25 persistent symptoms potentially associated with COVID-19 infection. The figure includes both the percentage reporting the symptom and the percentage of those who attributed the symptom to COVID-19. The most frequent persistent symptom overall was fatigue, reported by 55.4% and 48.0% of those (24.9% of the overall sample), attributed the fatigue directly to COVID-19. A total of nine symptoms were reported by 30% or more of respondents, and these included, in addition to fatigue, sleep disturbance, difficulties with attention, reduced quality of life, joint pain, difficulties with memory, depression, headache, and reduced daily functioning. For about half of the respondents who reported decreased quality of life or daily functioning these effects were attributed to COVID-19. For all the other symptoms the percentage was lower. There were several symptoms where the majority of those who reported the symptom also attributed it to COVID-19, at rates all greater than 67%. These included shortness of breath, reduced sense of smell, reduced sense of taste, and reduced lung function.

#### Factors Associated With Mental Health Outcomes

Table 2 includes correlations between the depression, anxiety and insomnia scores with participant background and health status variables. Alpha was set at *p* < .001, given the large sample size and large number of correlations calculated.

**Table 2:** Correlations Addressing Potential Predictors of Depression, Anxiety, and Insomnia

Potential Predictor	Depression (PHQ-9)	Anxiety (GAD-7)	Insomnia (ISI)
Age	-.17*	-.21*	-.04
Gender (female)	.07	.06	.09
Education (University Graduate)	-.06	-.04	-.09
Married or in a relationship	-.11*	-.04	.10*
Unemployed	.20*	.16*	.18*
City dwelling	.05	.05	.02
Children at home	-.03	.01	.02
Self-rated finance above average	-.15*	-.13*	-.07
Self-rated health above average	-.52*	-.43*	-.40*
History of a mental health condition	.33*	.35*	.25*
Current mental health condition	.55*	.57*	.35*
Infected with COVID-19	.15*	.10*	.13*
Risk factors for poor COVID-19 outcome^a^	.10*	.07	.12*
Worry about own health	.39*	.38*	.31*
Worry about others health	.28*	.34*	.26*
Worry about personal finance	.48*	.45*	.39*
Worry about world economy	.21*	.21*	.21*
Worry about the future	.45*	.41*	.34*
Worry total	.50*	.49*	.42*

*Note.* Correlations that reflect medium or large effect sizes in bold text.

^a^Sum of risk factors: age over 70, hypertension, angina, stroke, heart disease, diabetes, cancer, smoking, respiratory disease, and immune suppressant.

*p < .001.

There were several factors that were unrelated to the mental health outcomes, including gender, education, home setting (city versus suburbs or country), or having children at home. There were several other factors that achieved significant but small or inconsistent correlations, including age, relationship status, employment status, self-rated financial status, COVID-19 inflection, number of medical conditions increasing risk for poor COVID-19 outcomes, and worry about the world economy. Factors that achieved medium-sized or larger correlations included self-rated health, history of mental health problems, current mental health, and worries about own health, others health, personal finances, and the future. Amongst all of these, reported history of mental health problems was the strongest correlate.

Table 3 includes correlations between depression, anxiety, and insomnia with the 25 persistent symptoms. Every symptom correlated with all three outcomes at *p* < .001, except for changes in sense of smell which failed to significantly correlate with anxiety.

**Table 3:** Correlations of Persistent Symptoms During COVID-19 and Depression, Anxiety, and Insomnia

Symptom	Depression (PHQ-9)	Anxiety (GAD-7)	Insomnia (ISI)
Fatigue	.53*	.43*	.43*
Shortness of breath	.35*	.29*	.29*
Cough	.16*	.13*	.19*
Heart Palpitations	.35*	.33*	.26*
Weight Loss	.19*	.20*	.18*
Gut problems	.31*	.27*	.29*
Memory problems	.48*	.39*	.38*
Attention problems	.55*	.48*	.40*
Chest pain or pressure	.30*	.27*	.19*
Sleep difficulties	.49*	.45*	.67*
Skin changes	.21*	.17*	.16*
Changes to sense of taste	.14*	.09*	.11*
Changes to sense of smell	.11*	.07	.11*
Joint pain	.29*	.23*	.30*
Pins and needles in extremities	.29*	.27*	.26*
Nausea	.34*	.31*	.24*
Decreased lung function	.18*	.15*	.17*
Headache	.35*	.32*	.30*
Decreased appetite	.37*	.27*	.23*
Fever	.19*	.15*	.13*
Sore throat	.18*	.12*	.11*
Depression	.63*	.56*	.43*
Anxiety	.53*	.60*	.34*
Decrease quality of life	.51*	.41*	.36*
Decreased daily functioning	.46*	.36*	.36*
Total number of symptoms^a^	.59*	.49*	.48*

*Note.* Correlations that reflect medium or large effect sizes in bold text.

^a^Total number of symptoms here excludes items related to sleep, depression, anxiety, quality of life, and daily functioning.

*p < .001.

There were several symptoms that achieved medium or large correlations across all three outcomes. These included fatigue, memory problems, attention problems, sleeping difficulties, headache, depression, anxiety, decreased quality of life, decreased daily functioning, and the total number of symptoms.

### Multivariate Prediction Analyses

Three separate hierarchical multiple regression analyses were conducted with depression, anxiety, and insomnia as dependent variables (see Table 4). These were to examine the combined role of background, health status, COVID-19 infection, worry, and persistent post-COVID-19 symptoms in accounting for variance in these outcomes.

**Table 4:** Multiple Regression Analyses of Depression, Anxiety, and Insomnia

Block	Predictor	Dependent Variable					
		Depression		Anxiety		Insomnia	
		Δ*R*^2^	β	Δ*R*^2^	β	Δ*R*^2^	β
1	Background	.084**		.076**		.041**	
	Age		-.080**		-.11**		.010
	In a relationship		-.061**		-.00		-.068*
	Unemployed		.046		.027		.064*
	Finances above average		.023		.021		.067*
2	Health status	.27**		.21**		.17**	
	Health above average		-.17**		-.12**		-.11**
	Mental health history		.12**		.17**		.11**
	Physical risk factors		-.00		-.00		.00
3	COVID	.005*		.002		.005*	
	COVID infection		-.021		-.024		.010
4	Worry	.092**		.10**		.075**	
	Worry total		.23**		.26**		.23**
5	Persistent symptoms	.14**		.090**		.077**	
	Fatigue		.13**		.073*		.12**
	Shortness of breath		.078*		.069		.040
	Heart palpitations		.045		.094**		.010
	Gut problems		-.025		.010		.041
	Memory problems		.081*		.024		.067
	Attention problems		.20**		.21**		.054
	Chest pain/pressure		.035		.024		.050
	Joint pain		.039		.017		.076*
	Nausea		.065*		.078**		.010
	Headache		.081**		.094**		.075*
	Decreased appetite		.17**		.081**		.037
	Total number symptoms		.12		.12		.040
**Total *R*^2^**		**.59**		**.48**		**.37**	

*Note.* Regression coefficients that reflect significant effect sizes across all three dependent variables in bold text.

*p < .01. **p < .001.

Amongst these blocks of variables, history of COVID-19 infection was the least informative, accounting for less than 1% of variance in all cases. The four background variables, including age, relationship status, employment status, and self-rated financial status, accounted for 8.4%, 7.6%, and 4.1% of variance in depression, anxiety, and insomnia, respectively. Older age significantly predicted lower scores for depression and anxiety, and being single, unemployed, and, unexpectedly, being well off financially, each contributed significantly to higher score for insomnia, although the variance accounted for was modest.

The health status block including self-rated health, mental health history, and the summary score of physical conditions representing risks for poor COVID-19 accounted for 27.0%, 21.0%, and 17.0% of variance. Here the health rating and mental history were significant predictors, but the physical risk factors were not. The single best variable in the equations was the total COVID-related worry scores, accounting for 9.2%, 10.0%, and 7.5% of variance in the outcomes. With all other potential predictors included, on average the twelve persistent physical symptoms with medium effect sizes and the summary score for the number reported (adjusted to exclude depression, anxiety, sleeping problems or reduced quality of life or daily functioning) were relatively good predictors of outcomes as a set, accounting for 14.0% or variance in depression, 9.0% for anxiety, and 7.7% for insomnia. The specific symptoms most strongly related to the three outcomes were fatigue and headache, which both significantly predicted all three, and attention problems, which was a relatively strong predictor of both depression and anxiety, but not insomnia. Finally, we reran the regression analyses selecting only the participants who had reported a history of COVID-19 infection and the results did not change appreciably. For example, the variance accounted for by the persistent physical symptoms remained similar or the same, 14.0% for depression, 8.4% for anxiety, and 7.5% for insomnia.

## Discussion

>The purpose of this study was to examine levels of depression, anxiety, and insomnia, in Sweden at 18 months following the start of the pandemic and compare to our findings from an independent population sample in 2020. We also examined persistent COVID-19 symptoms in terms of prevalence and associations with the mental health outcomes, as well as risk factors known to increase vulnerability for mental ill health in the pandemic context (McCracken et al., 2020; Zvolensky et al., 2020). Compared to previous reports of mental health from the early pandemic, we found a decrease in level of depression and anxiety and an increase for insomnia. We found a high rate of persistent COVID-19 symptoms, with 84.5% of the sample reporting at least one persistent symptom, with fatigue being the most common. It appears that at the late phase of the pandemic, people in Sweden were still suffering from mental health problems, and from persistent symptoms. In addition, these persistent symptoms seemed to play a role in mental health over and above risk factors such as demographics, general health status, and COVID-19 related worry.

We found that 27% of our sample reported clinically significant levels of depression, whereas the rates for anxiety and insomnia were 16% and 45%, respectively. In our previous study (McCracken et al., 2020), the rates for the same mental health problems, were 30% for depression, 24% for anxiety, and 38% for insomnia. Although we observed a decrease for depression, this change is small and may be practically non-significant. On the other hand, the results for anxiety are in line with other reports indicating that the world is beginning to slowly recover from the mental health impacts associated with the pandemic (Manchia et al., 2022; Yarrington et al., 2021). The high rate of significant insomnia was unexpected, and the cause for this increase is unclear. In one study, insomnia during the pandemic was more common in women (Goncalves et al., 2022) and considering that 89.5% of our sample identified as women could be a factor. We note that the percentage meeting criteria as having significant insomnia is the same as the 44% who endorse sleeping problems in the list of persistent symptoms, the second most frequent complaint. Perhaps not surprisingly, our regression analyses showed that COVID-19-related worries were a strong predictor of insomnia.

The higher rates of depression, anxiety, and insomnia in Sweden are consistent with global prevalence of these identified in a systematic review and meta-analysis, calling prevalence rates “very high compared to normal times” (Mahmud et al., 2021) and with longitudinal data suggesting long term psychological disturbances in COVID-19 survivors (Mazza et al., 2022). Practically speaking, 67% of the participants meeting criteria for depression reported that this was having an impact on their daily functioning suggesting significant real-life consequences for the individual and society.

As a whole, the persistent symptoms assessed occurred very frequently, with 84.5% of the participants reporting at least one symptom lasting for at least six weeks, and there was a high rate of these symptoms even among those who reported no prior infection with COVID-19, 78%. Important to note is that some of the symptoms are likely entirely coincidental to the COVID-19 infection, but we have no clear way to specifically identify those that are and those that are not.

In our study, fatigue was the most common persistent symptom, with a prevalence of 55.4% and approximately half of these, 48%, attributing it directly to COVID-19. This finding corroborates consistent results across European studies on persistent COVID-19 symptoms, with prevalence rates of fatigue between 50-70% (Fernández-de-las-Peñas et al., 2021; Lopez-Leon et al., 2021). This is not to lose sight of the wider range of symptoms and that 30% of the participants reported having all of a total of nine symptoms including fatigue, sleep disturbances, difficulties with attention, reduced quality of life, joint pain, difficulties with memory, depression, headache and reduced daily functioning.

Looking at potential vulnerability factors, numerous participant characteristics were associated with at least one of the three mental health outcomes, including age, relationship status, employment status, financial status, risk for poor COVID-19 outcome, and worry about the world economy (see Table 3). Given the small correlations, practical implications for these results may be limited and findings should be replicated. However, factors that appeared more robustly linked to depression, anxiety and insomnia, presenting medium effect sizes, were self-rated overall health, history of mental health problems, current mental health status, and current worries. Amongst these, reported current mental health status was the strongest correlate.

The results from the multivariate analyses showed that history of COVID-19 infection was the least informative in explaining variance for depression, anxiety and insomnia, accounting for less than 1% of all three outcomes. Thus, contracting COVID-19 infection did not seem to impact on level of mental health in the pandemic context perhaps pointing to other contextual factors as more important. Background variables – age, relationship status, employment status, and self-rated financial status –, as a set, significantly predicted all of the three mental health outcomes, explaining 8.4%, 7.6%, and 4.1% of the variance in depression, anxiety, and insomnia, respectively. Older age seemed to function as a protective factor against increased levels of depression and anxiety. This also suggests that younger people are more vulnerable to mental ill health in the pandemic context, a result which has been well documented throughout the pandemic (McCracken et al., 2020; Pierce et al., 2020).

The health status variables, including, overall rated health, mental health history, and a summary score of physical conditions was the strongest predictor of risk for poor outcome in depression, anxiety and insomnia, accounting for close to half of the explained variance for each outcome. Overall health and mental history appeared similarly important whereas physical risk did not contribute at all. The relation between a previous history of mental ill health and higher levels of poor mental health in all outcomes, suggests increased vulnerability for mental ill health during the pandemic in those already burdened with the impacts of mental ill health

We found that the best single predictor of the mental health outcomes was COVID-19-related worry scores, accounting for 9.2%, 10.0%, and 7.5% of variance in depression, anxiety and insomnia. This finding may mean that for many people cognitive and emotional responses to the pandemic situation represent an underlying mechanism of the elevated levels of significant depression, anxiety, and insomnia.

Controlling for all other predictors, the 12 selected persistent symptoms and the summary score for the number reported (adjusted to exclude depression, anxiety, sleeping problems or reduced quality of life or daily functioning) accounted for 14.0% or variance in depression, 9.0% for anxiety, and 7.7% for insomnia. The symptoms most strongly related to the three outcomes were fatigue and headache, which both significantly predicted all three, and attention problems, which was a relatively strong predictor of both depression and anxiety, but not insomnia. We attempted to examine whether it mattered if the symptoms seemed to emerge directly from diagnosed COVID-19 exposure or not. We did this in two ways, by analyzing the COVID-19 attributions related to the symptoms and by analyzing the specific COVID-19 diagnosis groups. In either case it did not make any difference whether there was a direct link with COVID-19 infection or not. This may mean that these symptoms are not specific or uniquely linked to COVID-19 infection but may be caused by people’s experiences in the pandemic context, as mentioned before, in combination with other vulnerability factors. In fact, the nature of many of the symptoms included is that they are frequently associated with stress. These persistent symptoms are interesting, because their link with mental health outcomes seems stronger than their links with COVID-19 itself. We hasten to add that none of this is to deny the legitimacy of the symptoms nor to express an opinion on their genesis in any particular case.

One obvious limitation of this study is the relative absence of men, with 89.5% of participants identifying as women. This result was unexpected. One explanation may be related to the reported gender gap in COVID-19 risk perceptions, with men having lower estimates of their COVID-19 related risks compared to women (Lewis & Duch, 2021). This could lead to greater interest in pandemic research among women and, consequently, increased participation. Nevertheless, the uneven gender distribution limits what we can say about the experience of men. However, analyses of gender differences in mental ill health based on data from our previous pandemic survey (McCracken et al., 2020, 2021) showed no gender difference in the prevalence of the same mental health outcomes. Yet another unexpected result was the 100% vaccination rate in participants, leaving us unable to examine the role of this variable or to assume generalizability to those who are unvaccinated.

This survey, like our previous one (McCracken et al., 2020) is a cross sectional study and did not technically track the same people prospectively. There could be differences in the samples recruited that confound our comparison despite our intention to avoid these. Either way, we are unable to assess any direction in relations observed. Finally, we must acknowledge the inherent bias of self-report measures and the potential selective recruitment bias entailed through the use of social media. In fact, it may be this social media context that led to the recruitment of more women than men. In light of these limitations our results reported here need to be regarded as provisional.

To summarize, about 18 months after the pandemic began Swedish people continued to report a great deal of suffering and reduced health. Half of those who responded in this study appeared to suffer significant problems in at least one of the domains of depression, anxiety, or insomnia. In addition, many participants, 84.5%, reported one or more persistent symptom defined as potential effects of COVID-19. The most apparent risk factors for lower mental health included negative overall self-rated health, history of mental health problems, worries related to the pandemic, and persistent physical symptoms. It may be important to study whether symptoms and other negative health outcomes persist long term post pandemic, whether these represent a significant health burden, and might need to be addressed. In conclusion, this study emphasizes the need for comprehensive, long-term mental health strategies that address both psychological and physical health issues, particularly for vulnerable populations, as the world continues to recover from the COVID-19 pandemic.

## Data Availability

The data that support the findings of this study are available from the corresponding author upon reasonable request.

## Preregistration

The study was not preregistered.

## Reporting Guidelines

This article is written according to the JARS-QUANT guidelines.
